# Searching for cognitive enhancement in the Morris water maze: better and worse performance in D‐amino acid oxidase knockout (*Dao* ^−/−^) mice

**DOI:** 10.1111/ejn.13192

**Published:** 2016-03-23

**Authors:** David Pritchett, Amy M Taylor, Christopher Barkus, Sandra J Engle, Nicholas J Brandon, Trevor Sharp, Russell G Foster, Paul J Harrison, Stuart N Peirson, David M Bannerman

**Affiliations:** ^1^ Nuffield Department of Clinical Neurosciences (Nuffield Laboratory of Ophthalmology)John Radcliffe HospitalUniversity of OxfordOxfordUK; ^2^ Department of Experimental PsychologyUniversity of OxfordTinbergen Building, 9 South Parks Road, OxfordOX1 3UD, UK; ^3^ Department of PharmacologyUniversity of OxfordOxfordUK; ^4^ Pfizer Inc.GrotonCTUSA; ^5^ Department of PsychiatryWarneford HospitalUniversity of OxfordOxfordUK; ^6^ Present address: AstraZeneca Neuroscience iMED141 Portland StreetCambridge, MA 02139USA

**Keywords:** D‐serine, glutamate, *N*‐methyl‐d‐aspartate receptor, pre‐clinical model, schizophrenia

## Abstract

A common strategy when searching for cognitive‐enhancing drugs has been to target the *N*‐methyl‐d‐aspartate receptor (NMDAR), given its putative role in synaptic plasticity and learning. Evidence in favour of this approach has come primarily from studies with rodents using behavioural assays like the Morris water maze. D‐amino acid oxidase (DAO) degrades neutral D‐amino acids such as D‐serine, the primary endogenous co‐agonist acting at the glycine site of the synaptic NMDAR. Inhibiting DAO could therefore provide an effective and viable means of enhancing cognition, particularly in disorders like schizophrenia, in which NMDAR hypofunction is implicated. Indirect support for this notion comes from the enhanced hippocampal long‐term potentiation and facilitated water maze acquisition of ddY/*Dao* ^−^ mice, which lack DAO activity due to a point mutation in the gene. Here, in *Dao* knockout (*Dao* ^−/−^) mice, we report both better and worse water maze performance, depending on the radial distance of the hidden platform from the side wall of the pool. *Dao* ^−/−^ mice displayed an increased innate preference for swimming in the periphery of the maze (possibly due to heightened anxiety), which facilitated the discovery of a peripherally located platform, but delayed the discovery of a centrally located platform. By contrast, *Dao* ^−/−^ mice exhibited normal performance in two alternative assays of long‐term spatial memory: the appetitive and aversive Y‐maze reference memory tasks. Taken together, these results question the proposed relationship between DAO inactivation and enhanced long‐term associative spatial memory. They also have generic implications for how Morris water maze studies are performed and interpreted.

## Introduction

The search for cognitive‐enhancing drugs is a primary goal in industry, medicine and academia, which has enormous therapeutic relevance for numerous psychiatric and neurological disorders. A common strategy has been to target the *N*‐methyl‐d‐aspartate subtype of glutamate receptor, given its putative role in certain forms of synaptic plasticity, such as hippocampal long‐term potentiation, and in learning and memory (Martin *et al*., 2000; Silva, 2003). Evidence in support of this approach has come primarily from studies with rodents, using behavioural assays such as the Morris water maze (see Morris, 1984; D'Hooge & De Deyn, 2001; Vorhees & Williams, 2006). This task can measure hippocampus‐dependent long‐term spatial memory, and involves training rodents to find a hidden platform in a fixed location in order to escape from lukewarm water.

There has been considerable interest in the possibility that targeting the glycine co‐agonist site on the *N*‐methyl‐d‐aspartate receptor (NMDAR) complex, either directly or indirectly, might provide an effective and viable means of enhancing cognition (see Labrie & Roder, 2010; Javitt, 2012; Hashimoto, 2014). It is now widely acknowledged that glycine and D‐serine are the primary endogenous co‐agonists of extra‐synaptic and synaptic NMDARs, respectively (Schell *et al*., 1995; Mothet *et al*., 2000; Oliet & Mothet, 2009; Papouin *et al*., 2012). Drugs that inhibit D‐amino acid oxidase (DAO), an enzyme that degrades D‐serine, are therefore under investigation as potential cognitive enhancers (Smith *et al*., 2010; Ferraris & Tsukamoto, 2011; Sacchi *et al*., 2013). This approach may be particularly relevant to schizophrenia, which is thought to involve NMDAR hypofunction (Olney *et al*., 1999; Kantrowitz & Javitt, 2010; Marek *et al*., 2010; Coyle, 2012); moreover, DAO has been implicated in this hypofunction (Verrall *et al*. 2010).

As proof of principle, *Dao* knockout (*Dao* ^−/−^) mice have been generated and subjected to tests of cognition. *Dao* ^−/−^ mice exhibit improved spatial and non‐spatial short‐term memory (Pritchett *et al*., 2015), and improved object recognition memory has also been reported in wild‐type (WT) rats following the administration of a DAO inhibitor (Hopkins *et al*., 2013). However, the relationship between DAO function and associative, long‐term spatial memory is less clear. Enhanced hippocampal long‐term potentiation and facilitated water maze acquisition have been reported in the ddY/*Dao* ^−^ mouse, which lacks DAO activity due to a point mutation in the gene (Maekawa *et al*., 2005), but water maze acquisition is unaffected in the *Dao1* ^*G181R*^ mouse, a related *Dao* mutant (Labrie *et al*., 2009). To date, the long‐term spatial memory of the *Dao* ^−/−^ mouse has not been evaluated.

In the present study, we assessed the long‐term spatial memory of *Dao* ^−/−^ mice in three separate paradigms: the Morris water maze, appetitive Y‐maze task, and aversive Y‐maze swim‐escape task. Collectively, our data suggest that long‐term spatial memory is unaltered in *Dao* ^−/−^ mice. More generally, they raise important issues concerning the potentially confounding effect of radial platform distance on task performance in the Morris water maze.

## Materials and methods

### Animals

The *Dao* ^−/−^ mice were generated as described previously (Rais *et al*., 2012). Heterozygous mice were mated to produce *Dao* ^−/−^ mice and WT littermate controls. *Dao* ^−/−^ mice have undetectable levels of DAO mRNA and immunoreactivity (Betts *et al*., 2014; Schweimer *et al*., 2014). Mice were at least 7 weeks old at the onset of behavioural testing. Prior to behavioural testing, mice were handled for 2 min per day for five consecutive days. Mice were housed under a 12 : 12 h light/dark cycle with lights on at 07 : 00 h, with access to food and water *ad libitum* unless otherwise stated. Mice were singly‐housed to enable comparison with previous work, which included circadian assays that necessitated single housing (Pritchett *et al*., 2015). All testing was conducted between 10 : 00 and 17 : 00 h. All behavioural procedures were performed in accordance with the United Kingdom Animals (Scientific Procedures) Act of 1986 and the University of Oxford Policy on the Use of Animals in Scientific Research. All experiments were approved by the University of Oxford Animal Welfare and Ethical Review Board. Separate cohorts of experimentally naive mice were used in each of Experiments 1, 2, 3 and 5. The mice used in Experiment 3 (unconditioned anxiety in the anxiogenic open field test) were subsequently tested in Experiment 4 (aversively motivated Y‐maze swim‐escape task).

### Experiments 1 and 2: spatial memory in the Morris water maze

Male and female mice were trained on the spatial reference memory version of the Morris water maze hidden platform task to assess their associative, long‐term spatial memory. Testing was based on an established hippocampus‐dependent protocol (Deacon *et al*., 2002; Reisel *et al*., 2002; Bannerman *et al*., 2012). The water maze was 2 m in diameter, with 90‐cm‐high walls. The water level was 65 cm and the water temperature was 20 °C ± 2 °C. The platform was 20 cm in diameter and positioned 1.5 cm beneath the water surface. White paint was added to the water to obscure the platform from view. A variety of distal extra‐maze cues were positioned around the laboratory (e.g. wall posters, racks of equipment, lights). All training trials (during acquisition and reversal) were 90 s in duration. Mice that did not locate the platform within this time were guided towards the platform by the experimenter.

**Figure 1:**
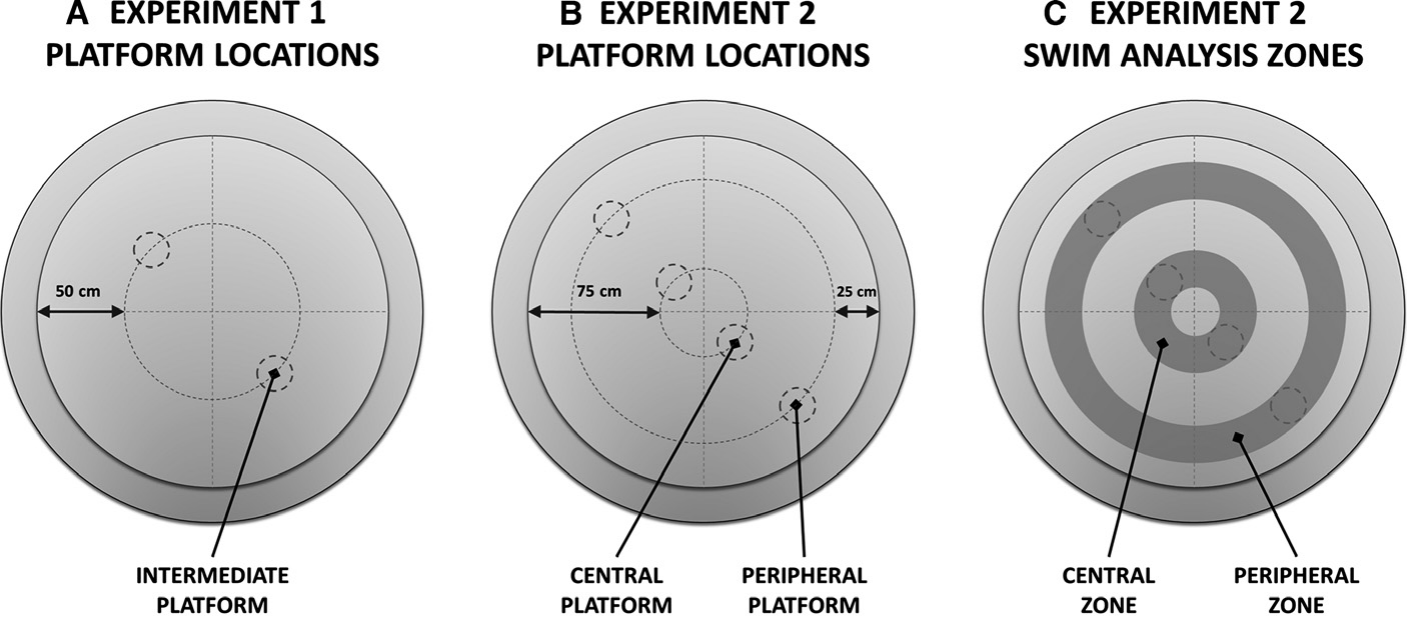
Morris water maze platform locations and swim analysis zones. (A) In Experiment 1, the hidden platform was located 50 cm from the side wall of the pool, in either the NW or SE quadrant. (B) In Experiment 2, the hidden platform was located either 75 or 25 cm from the side wall (henceforth referred to as the central and peripheral platform locations, respectively), in either the NW or SE quadrant. (C) Using the software package ANY‐maze 4.5 (Stoelting, Wood Dale, IL, USA), the pool was divided into nominal central and peripheral zones, encompassing the central and peripheral platform locations. Percent time spent in each of these zones was computed for each mouse for each trial of Experiment 2.

Mice were given a single training session each day, consisting of four consecutive trials, with an inter‐trial interval of 45 s (30 s spent on the platform and 15 s drying time). The platform was located in either the NW or SE quadrant, with platform location counterbalanced across genotype groups. The platform was located at different radial distances from the side wall (see Fig. 1). In Experiment 1, the platform was located 50 cm from the side wall. In Experiment 2, the platform was located either 25 or 75 cm from the side wall. Probe trials were also conducted to assess spatial memory performance, during which the platform was removed and mice were able to swim freely for 60 s. In all trials, mice were released at the edge of the pool, facing towards the wall. The release point was varied pseudo‐randomly across trials and test sessions, but the same sequence was used for all animals. Trials were recorded with a CCTV camera (WV‐BP334, Panasonic, Osaka, Japan) suspended above the pool, and automated tracking was conducted using the software package Watermaze 3.31 (Actimetrics, IL, USA). The dependent variables were escape path length (m) and escape latency (s) for training trials. For probe trials, the dependent variables were platform crosses, platform crossing accuracy score, and percent time spent in the training (platform) quadrant (i.e. the quadrant in which the platform was located during training). Platform crosses were automatically registered whenever a mouse entered the 20‐cm diameter zone in which the platform was previously located. The platform crossing accuracy score = TRA−[(OPP + ADJL + ADJR)/3], where TRA, OPP, ADJL, and ADJR are the number of platform crossings in the training, opposite, adjacent left, and adjacent right quadrants, respectively (Bannerman *et al*., 2008).

### Experiment 3: unconditioned anxiety in the anxiogenic open field test

Male mice were assessed in a brightly lit open field test of anxiety (Line *et al*., 2011; De Filippis *et al*., 2015). The apparatus was a white metal cylinder, with a diameter of 60 cm and a height of 60 cm. Illumination of the apparatus was homogeneous, measuring 2000 lux at the base of the arena. Mice were released at the edge of the arena, facing towards the wall, and allowed to explore the apparatus for 1 min. Trials were recorded with a near‐infrared CCTV camera (Maplin Electronics, Rotherham, UK) positioned above the apparatus, and automated tracking was performed using ANY‐maze 4.5 (Stoelting, Wood Dale, IL, USA). Using this software, the apparatus was divided into nominal peripheral and central zones. The peripheral zone was 6 cm wide, and the central zone was 48 cm in diameter. The animal's entire body was tracked, and its position within the apparatus was determined on a frame‐by‐frame basis. A zone entry was deemed to have occurred when at least 90% of the animal's body had entered the zone in question.

### Experiment 4: spatial memory in the aversively motivated Y‐maze swim‐escape task

Male mice were trained on another aversively motivated, spatial reference memory test, in the form of the Y‐maze swim‐escape task (Lyon *et al*., 2011). Like the Morris water maze, performance in this task relies on the ability to associate a particular spatial location with the hidden escape platform, but performance is unlikely to be influenced by innate differences in preferred swimming distance from the side walls. The apparatus was a transparent acrylic Y‐maze, with each arm measuring 30 cm in length and 9 cm in width. The maze was surrounded by a 21‐cm‐high wall. The maze was filled with water, rendered opaque with white paint, to a depth of 12 cm. The water temperature was 20 °C ± 2 °C. A variety of distal extra‐maze cues were positioned around the laboratory. As in the Morris water maze task, mice could escape from the water by climbing onto a hidden platform positioned 1.5 cm beneath the water surface. The platform was positioned at the end of one of the three arms, with the choice of target arm counterbalanced across genotype groups. In all trials, mice were released at the end of one of the two non‐target arms, facing towards the wall. The release arm was varied pseudo‐randomly across trials and test sessions, but the same sequence was used for all animals. Training trials were 90 s in duration, and mice that did not locate the platform within this time were guided towards the platform by the experimenter. All mice completed a single training session each day for six consecutive days. Each session consisted of five consecutive trials, with an inter‐trial interval of 45 s (30 s spent on the platform and 15 s drying time). During each training trial, choice accuracy (i.e. whether the first arm entered was the target arm) was recorded. Data were analysed in blocks of 10 trials.

A probe trial was performed on day 7, during which mice were able to swim freely for 60 s with the platform removed. Probe trials were recorded with a near‐infrared CCTV camera (Maplin Electronics) suspended above the apparatus, and automated tracking was conducted using ANY‐maze 4.5 (Stoelting). The percent time spent in each of the three maze arms was recorded.

To confirm that the aversively motivated Y‐maze swim‐escape task reflected hippocampus‐dependent spatial memory, separate groups of sham and hippocampal‐lesioned mice were tested in the same apparatus (Fig. S4). Hippocampal lesions were made by stereotaxic injection of the excitotoxin *N*‐methyl‐d‐aspartate, in a similar manner to that described previously (e.g. for details, see Deacon *et al*., 2002; Bannerman *et al*., 2012).

### Experiment 5: spatial memory in the appetitively motivated Y‐maze task

Male mice were trained on a dry land, appetitively motivated, spatial reference memory Y‐maze task, which we have shown previously requires the hippocampus (Deacon *et al*., 2002; Reisel *et al*., 2002). The design was broadly analogous to that of the Y‐maze swim‐escape task, except that mice ran to receive a food reward (sweetened condensed milk), rather than swam to escape from water. The apparatus was a black wooden Y‐maze, with each arm measuring 50 cm in length and 9 cm in width. The maze was surrounded by a 1‐cm‐high wall. The maze was elevated 80 cm above the ground. Mice were maintained at 85–90% of their free‐feeding weight throughout testing. In each trial, a mouse was released from one of the two non‐baited arms and allowed to enter one of the other two arms. Mice that selected the baited arm were allowed to consume the milk reward before being returned to their home‐cage, whereas mice that entered the incorrect arm were immediately returned to the home‐cage. All mice completed a single training session each day for 10 consecutive days. Each session consisted of five trials, with an inter‐trial interval of approximately 5 min. During each training trial, choice accuracy (i.e. whether the first arm entered was the target arm) was recorded. Data were analysed in blocks of 10 trials. On days 11 and 12, mice completed a session of five post‐choice bait trials (i.e. 10 trials in total). In these trials, the milk reward was not added to the target arm until after the mouse had entered this arm; this ensured that mice could not solve the task based on the sight or smell of the milk reward.

### Data analysis

All statistical analyses were performed with SPSS 22.0 (IBM, Armonk, New York, NY, USA). Unless otherwise stated, all reported statistics are the result of anovas, with genotype as the principal independent variable, in addition to sex, day, trial number (within a given training session), and radial platform distance where applicable. Significant interactions were explored further by analysis of simple main effects. Differences were considered to be statistically significant at *P*‐values < 0.05.

## Results

### Experiment 1: enhanced Morris water maze performance in Dao^−/−^ mice

**Figure 2:**
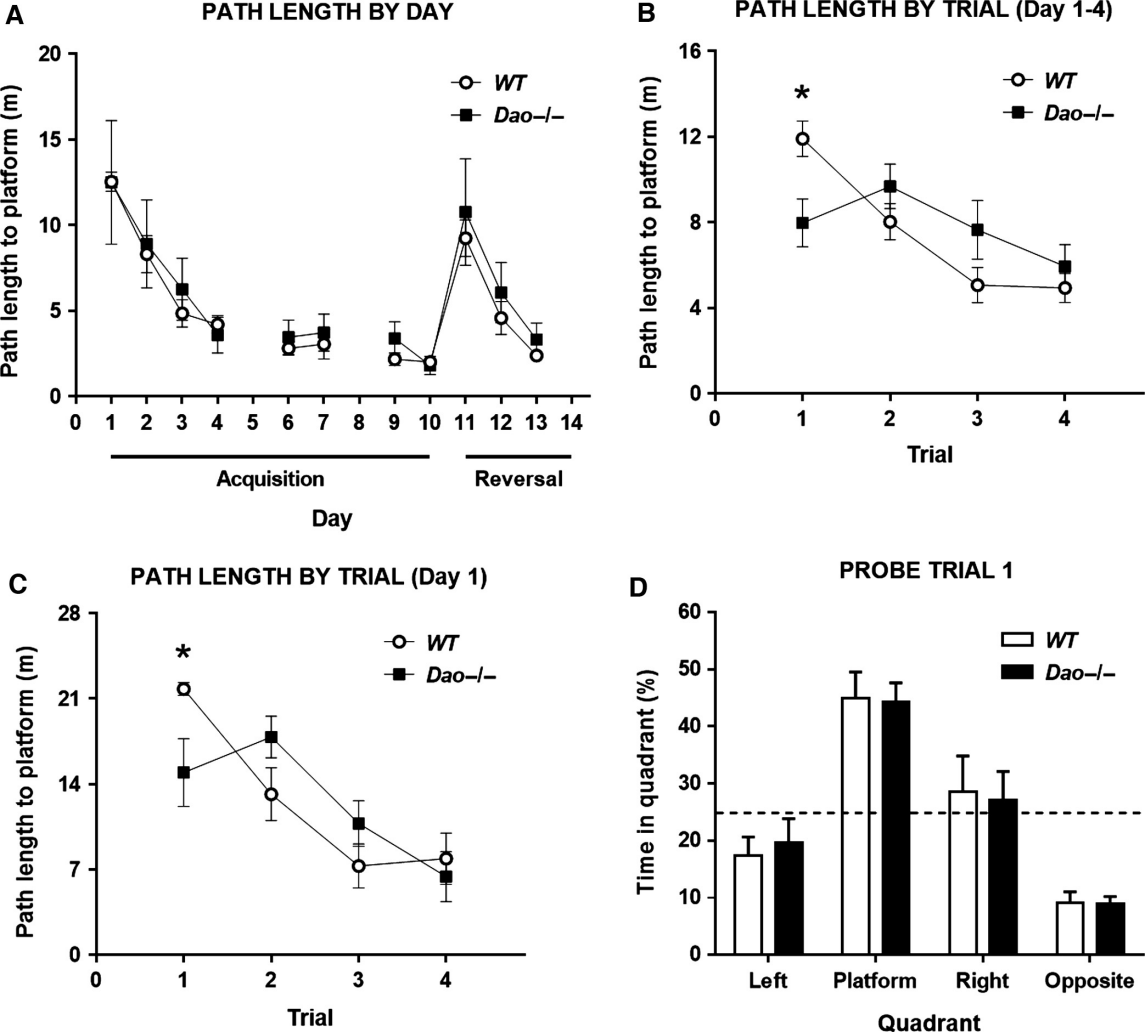
Enhanced Morris water maze performance in *Dao* ^−/−^ mice. A total of 24 mice (*n *= 6 males and *n *= 6 females per genotype) participated in Experiment 1. (A) With data collapsed across the four trials of each training session, genotype had no apparent impact on water maze performance (path length to platform) during the acquisition phase or reversal learning phase. (B) Across the first 4 days of training, however, *Dao* ^−/−^ mice significantly outperformed WT mice on the first trial of each day (path length to platform), but not on the subsequent three trials, yielding a significant interaction between genotype and trial number (*P* = 0.002). The graph shows path length to the platform in the four trials of a session (Day), collapsed across days 1–4. (C) The same pattern of performance was evident on the very first day of training, again yielding a significant genotype × trial number interaction (*P* = 0.020). The graph shows path length to the platform in the four trials of day 1. (D) Genotype had no effect on probe trial 1 performance. The graph shows percent time spent in each quadrant of the pool (platform indicates the quadrant in which the platform was located during training; other quadrants are defined relative to this quadrant). *P‐value < 0.05. Error bars depict the SEM. Dashed line, chance performance (25%).

Experimentally naive WT and *Dao* ^−/−^ mice were trained on the standard, fixed location, hidden escape platform, spatial reference memory version of the Morris water maze task. The platform was located 50 cm from the side wall. All mice swam well in the pool and there was very little evidence of floating behaviour. Across the first 4 days of training, there were main effects of day and trial number for both path length and latency (all *P*‐values ≤ 0.001; Fig. 2A and B, and Supporting Information Fig. S1A and B), in addition to day × trial number interactions (both *P*‐values ≤ 0.023; data not shown), reflecting acquisition of the task. By contrast, there were no overall main effects of genotype, nor any genotype × day interactions (all *P*‐values ≥ 0.654).

Intriguingly, however, *Dao* ^−/−^ mice performed consistently better than WT controls on the first trial of each day (Fig. 2B and Fig. S1B). Analysis revealed a genotype × trial number interaction for both path length (*F*_3,60_ = 5.594, *P* = 0.002; Fig. 2B) and latency (*F*_3,60_ = 5.566, *P* = 0.002; Fig. S1B) across the first 4 days of training. Subsequent analysis of simple main effects confirmed that *Dao* ^−/−^ mice had outperformed WT controls on the first trial of each day (path length: *F*_1,20_ = 7.305, *P* = 0.014; latency: *F*_1,20_ = 8.517, *P* = 0.008; Fig. 2B and Fig. S1B), but that the two groups had performed similarly on trials 2, 3 and 4 (all *P*‐values ≥ 0.133).

Given that performance on the first trial of each day was dependent on memory lasting at least 24 h, the data from Experiment 1 are potentially consistent with the previous report of enhanced long‐term memory acquisition in the Morris water maze in ddY/*Dao* ^−^ mutant mice (Maekawa *et al*., 2005). However, closer inspection of the data revealed that the genotype × trial number interaction remained even when day 1 was considered in isolation (path length: *F*_3,60_ = 3.537, *P* = 0.020; Fig. 2C, latency: *F*_3,60_ = 3.098, *P* = 0.033; Fig. S1C). Thus, *Dao* ^−/−^ mice significantly outperformed WT controls on the very first trial of the very first day of water maze training (path length: *F*_1,20_ = 5.393, *P* = 0.031; latency: *F*_1,20_ = 5.733, *P* = 0.027), at which point they would have learned little, if anything, about the spatial location of the platform.

On day 5 (i.e. after 4 days of training), a probe trial was performed as an additional test of spatial memory. In this probe trial, both groups showed a strong preference for the training quadrant (where the platform was normally located), and genotype had no effect on platform crosses (*F*_1,19_ = 0.803, *P* = 0.381), platform crossing accuracy score (*F*_1,19_ = 0.615, *P* = 0.443), latency to the first platform cross (*F*_1,19_ = 3.185, *P* = 0.090), or percent time spent in the training (platform) quadrant (*F*_1,19_ = 0.074, *P* = 0.789; Fig. 2D). Note that one *Dao* ^−/−^ mouse was discounted from these analyses, as it remained motionless for the entirety of the probe trial.

A further 4 days of training (days 6–7 and 9–10) yielded main effects of day for both path length and latency (both *P*‐values ≤ 0.035; Fig. 2A and Fig. S1A), but there were no additional effects or interactions involving genotype (all *P*‐values ≥ 0.081; Fig. 2A and Fig. S1A). Likewise, in a second probe trial performed on day 8, genotype had no effect on platform crosses (*F*_1,20_ = 1.263, *P* = 0.274), platform crossing accuracy score (*F*_1,20_ = 2.006, *P* = 0.172), latency to the first platform cross (*F*_1,20_ = 1.048, *P* = 0.318), or percent time spent in the training (platform) quadrant (*F*_1,20_ = 0.716, *P* = 0.407; Fig. S1D). The performance of the two groups was also indistinguishable when the platform was moved to a novel spatial location in the diametrically opposite quadrant of the pool. This 3‐day reversal learning phase (days 11–13) yielded main effects of day and trial number for both path length and latency (all *P*‐values < 0.001; Fig. 2A and Fig. S1A), in addition to day × trial number interactions (both *P*‐values < 0.001; data not shown), reflecting acquisition of the novel platform location. Again, however, there were no effects or interactions involving genotype (all *P*‐values ≥ 0.110; Fig. 2A and Fig. S1A). The spatial memory performance of the two groups was also well matched in a final probe trial that was performed on day 14, after 3 days of reversal training to a new platform location in the opposite quadrant; genotype had no effect on platform crosses (*F*_1,20_ = 0.062, *P* = 0.806), platform crossing accuracy score (*F*_1,20_ = 0.031, *P* = 0.863), latency to the first platform cross (*F*_1,20_ = 0.009, *P* = 0.926), or percent time spent in the training (platform) quadrant (*F*_1,20_ = 0.982, *P* = 0.334; Fig. S1E).

The *Dao* ^−/−^ mice swam consistently faster than WT mice throughout the acquisition phase (*F*_1,20_ = 5.345, *P* = 0.032; data not shown). However, this cannot explain the enhanced first trial performance of the *Dao* ^−/−^ mice, as their superiority was evident in both the latency and path length data. Genotype had no effect on swim speed during the reversal learning phase (*F*_1,20_ = 0.009, *P* = 0.926; data not shown). There were no interactions between genotype and sex for any of the performance measures in Experiment 1.

### Experiment 2: water maze performance in Dao^−/−^ mice depends on the radial distance of the hidden platform from the side wall

It is theoretically possible that *Dao* ^−/−^ mice were better at remembering which areas of the pool they had already visited as the first trial of day 1 progressed, thereby increasing their search efficiency. However, an alternative explanation for their immediate performance advantage is that *Dao* ^−/−^ mice have an innate tendency to swim in particular zones of the pool (as defined by the radial distance from the side wall), rendering them more likely to encounter the escape platform simply by chance. This hypothesis was explored in Experiment 2.

**Figure 3:**
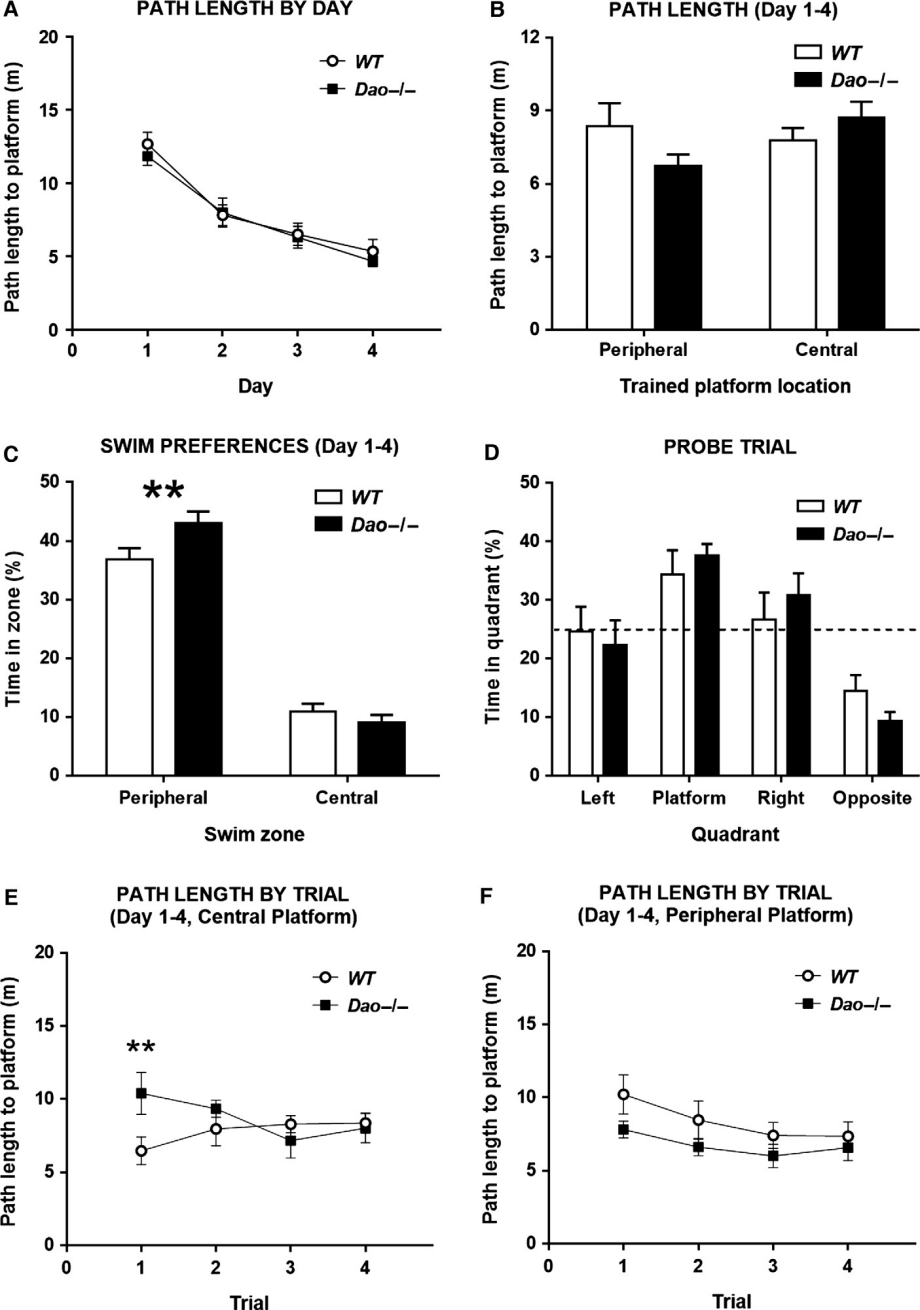
Water maze performance in *Dao* ^−/−^ mice depends on the radial distance of the hidden platform from the side wall. A total of 46 mice (*n *= 11 males and *n *= 12 females per genotype) participated in Experiment 2. (A) With data collapsed across the four trials of each training session, and across the two radial platform distances, genotype had no apparent impact on water maze performance (path length to platform) during the 4‐day acquisition phase. (B) The performance (mean path length to platform across all 16 acquisition trials) of *Dao* ^−/−^ mice was numerically superior to that of WT mice in the peripheral platform group, but numerically inferior in the central platform group, yielding a borderline‐significant interaction between genotype and radial platform distance (*P* = 0.05). (C) *Dao* ^−/−^ mice spent significantly more time than WT mice in the peripheral zone (see Fig. 1C) of the pool. (D) Genotype had no effect on probe trial performance. The graph shows percent time spent in each quadrant of the pool (platform indicates the quadrant in which the platform was located during training; other quadrants are defined relative to this quadrant). (E) Across the 4 days of training, WT mice in the central platform condition significantly outperformed *Dao* ^−/−^ mice on trial 1 (path length to platform), but not on the subsequent three trials, yielding a significant interaction between genotype and trial number (*P* = 0.022). (F) By contrast, there was no genotype × trial number interaction for path length to platform in the peripheral platform condition. **P‐value < 0.01. Error bars depict the SEM. Dashed line, chance performance (25%).

Separate groups of experimentally naive *Dao* ^−/−^ and WT mice were trained to locate platforms at different radial distances from the side wall. For half of the animals, the platform was positioned 25 cm from the side wall (peripheral location), and for the other half it was positioned 75 cm from the side wall (central location). Mice completed four trials per day for 4 days. Strikingly, *Dao* ^−/−^ mice outperformed WT mice in the peripheral platform condition, but were outperformed by WT mice in the central platform condition (Fig. 3B and Fig. S2B).

Across the 4 days of training, there were main effects of day for both path length and latency (path length: *F*_3,114_ = 46.328, *P* < 0.001; latency: *F*_3,114_ = 43.846, *P* < 0.001; Fig. 3A and Fig. S2A), reflecting acquisition of the task. By contrast, there were no overall main effects of genotype, nor any genotype × day interactions (all *P*‐values ≥ 0.480). Critically, however, there were borderline‐significant interactions between genotype and radial platform distance (path length: *F*_1,38_ = 4.020, *P* = 0.05; latency: *F*_1,38_ = 4.263, *P* = 0.046). These interactions reflected the fact that path lengths and latencies were shorter in *Dao* ^−/−^ mice than WT mice in the peripheral platform condition, but longer in *Dao* ^−/−^ mice than WT mice in the central platform condition, although none of the direct post‐hoc comparisons reached statistical significance (peripheral condition path length: *F*_1,20_ = 2.400, *P* = 0.137; peripheral condition latency: *F*_1,20_ = 3.268, *P* = 0.086; central platform path length: *F*_1,18_ = 1.745, *P* = 0.203; central platform latency: *F*_1,18_ = 1.188, *P* = 0.290; Fig. 3B and Fig. S2B).

Importantly, there were also significant three‐way interactions between genotype, trial number and radial platform distance (path length: *F*_3,114_ = 3.368, *P* = 0.021; latency: *F*_3,114_ = 3.128, *P* = 0.029). As in Experiment 1, the discrepancy between *Dao* ^−/−^ mice and WT mice was greatest on the first trial of each day (Fig. 3E, Fig. 3F and Fig. S2E). In the central platform group, *Dao* ^−/−^ mice were outperformed by WT controls on the first trial of each day (path length: *F*_1,18_ = 8.184, *P* = 0.010; latency: *F*_1,18_ = 6.061, *P* = 0.024), but the two groups performed similarly on trials 2, 3 and 4 (all *P*‐values ≥ 0.208). Consequently, there were genotype × trial number interactions in the central platform condition (path length: *F*_3,54_ = 3.495, *P* = 0.022; latency: *F*_3,54_ = 3.074, *P* = 0.035; Fig. 3E and Fig. S2E). By contrast, there were no genotype × trial number interactions in the peripheral platform condition (path length: *F*_3,60_ = 0.441, *P* = 0.724; latency: *F*_3,60_ = 0.484, *P* = 0.694; Fig. 3F and Fig. S2F).

As in Experiment 1, the altered performance of the *Dao* ^−/−^ mice was evident on the first trial of the first day, when the mice knew little, if anything, about the spatial location of the platform. Hence, there were also interactions between genotype and radial platform distance when trial 1 of day 1 was analysed in isolation (path length: *F*_1,38_ = 4.997, *P* = 0.031; latency: *F*_1,38_ = 5.419, *P* = 0.025). Analysis of simple main effects revealed that these significant interactions reflected the fact that path lengths and latencies were significantly longer in *Dao* ^−/−^ mice than WT mice in the central platform condition (path length: *F*_1,18_ = 5.180, *P* = 0.035; latency: *F*_1,18_ = 4.501, *P* = 0.048), but numerically (although not significantly) shorter in *Dao* ^−/−^ mice than WT mice in the peripheral platform condition (path length: *F*_1,20_ = 0.942, *P* = 0.343; latency: *F*_1,20_ = 1.460, *P* = 0.241; Fig. S2C and D).

To directly test whether the differential performance of WT and *Dao* ^−/−^ mice in the central and peripheral platform conditions was a reflection of different swim/search tendencies, we analysed the percentage of time spent in the peripheral and central zones of the pool, respectively, across the 16‐trial acquisition phase. The locations of both zones are defined in the Materials and methods. *Dao* ^−/−^ mice spent significantly more time than WT mice in the peripheral zone (*F*_1,38_ = 8.097, *P* = 0.007), but numerically (although not significantly) less time than WT mice in the central zone (*F*_1,38_ = 2.735, *P* = 0.106; Fig. 3C), irrespective of whether the platform was positioned in a central or peripheral location.

As in Experiment 1, spatial memory performance in the two genotype groups was well matched after 4 days of training, as demonstrated by their performance in a probe trial conducted on day 5. Genotype had no effect on platform crosses (*F*_1,38_ = 0.915, *P* = 0.345), platform crossing accuracy score (*F*_1,38_ = 0.129, *P* = 0.721), latency to the first platform cross (*F*_1,38_ = 0.445, *P* = 0.509), or percent time spent in the training (platform) quadrant (*F*_1,38_ = 0.483, *P* = 0.492; Fig. 3D). There were no interactions between radial platform distance and genotype (both *P*‐values ≥ 0.702), or indeed any other interactions (all *P*‐values ≥ 0.103) for probe trial performance. In contrast to Experiment 1, genotype had no effect on swim speed in Experiment 2 (*F*_1,38_ = 1.571, *P* = 0.218; data not shown).

In Experiment 2, the effect of genotype and radial platform distance on water maze performance was present in males but not females. This was reflected in a significant three‐way interaction between genotype, radial platform distance and sex for path length to the platform (path length: *F*_1,38_ = 4.409, *P* = 0.042; latency: *F*_1,38_ = 3.345, *P* = 0.075). Further analysis with separate anovas for each sex confirmed that, across the 16‐trial acquisition phase, interactions between genotype and radial platform distance were clearly evident in males (path length: *F*_1,18_ = 5.728, *P* = 0.028; latency: *F*_1,18_ = 5.025, *P* = 0.038; Fig. S3A‐D), but completely absent in females (path length: *F*_1,20_ = 0.007, *P* = 0.932; latency: *F*_1,20_ = 0.048, *P* = 0.828; Fig. S3E‐H). However, there was no main effect of sex on time spent in the peripheral zone or central zone of the pool (*P*‐values ≥ 0.215), and no interaction between genotype and sex for either variable (*P*‐values ≥ 0.423).

### Experiment 3: increased anxiety in Dao^−/−^ mice in the anxiogenic open field test

**Figure 4:**
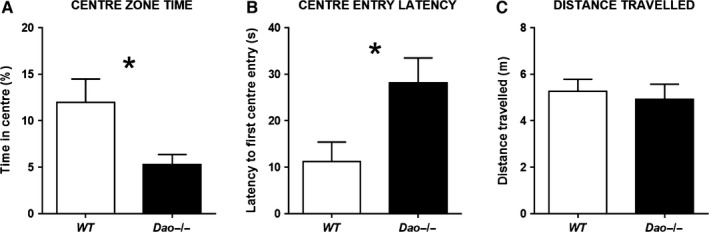
Increased anxiety in *Dao* ^−/−^ mice in the anxiogenic open field test. A total of 28 male mice (*n *= 14 per genotype) participated in Experiment 3. Relative to WT mice, *Dao* ^−/−^ mice (A) spent less time in the central zone of the arena, and (B) took longer to make their first centre zone entry. (C) Distance travelled did not differ between *Dao* ^−/−^ and WT mice. *P‐value < 0.05. Error bars depict the SEM.

To evaluate whether the different swim/search tendencies of WT and *Dao* ^−/−^ mice could reflect differences in emotionality, we assessed anxiety in a brightly lit, circular open field test in experimentally naive mice. We have previously demonstrated that male and female *Dao* ^−/−^ mice exhibit similarly increased levels of anxiety in a number of ethological, unconditioned anxiety tests, including in a different open field test in a rectangular arena with black walls (Pritchett *et al*., 2015). In the present study, male *Dao* ^−/−^ mice were more anxious than their WT littermates; they spent less time than WT mice in the centre zone of the arena (*F*_1,26_ = 5.945, *P* = 0.022; Fig. 4A), and took longer to make their first centre zone entry (*F*_1,26_ = 6.132, *P* = 0.020; Fig. 4B). This increased anxiety‐like behaviour of *Dao* ^−/−^ mice was not an artefact of reduced locomotor activity, as genotype had no effect on total distance travelled (*F*_1,26_ = 0.099, *P* = 0.756; Fig. 4C).

### Experiment 4: normal spatial memory in Dao^**−/−**^ mice in the aversively motivated Y‐maze swim‐escape task

**Figure 5:**
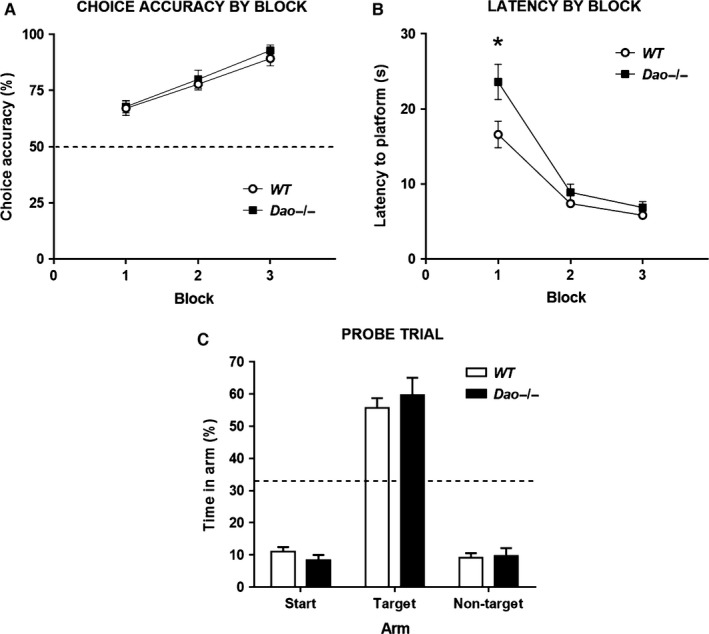
Normal spatial memory in *Dao* ^−/−^ mice in the aversively motivated Y‐maze swim‐escape task. A total of 28 male mice (*n *= 14 per genotype) participated in Experiment 4. (A) Genotype had no effect on choice accuracy (% correct trials) in this task. (B) *Dao* ^−/−^ mice took longer than WT mice to reach the hidden escape platform, particularly on the first block of training. (C) Genotype had no effect on probe trial performance (% time spent in the target arm of the Y‐maze, the arm in which the platform was located during training). *P‐value < 0.05. Error bars depict the SEM. Dashed line, chance performance (50% for acquisition phase and 33.3% for probe trial).

After completion of the open field test, the same cohort of mice was subjected to the aversively motivated, swim‐escape version of the Y‐maze reference memory task. The arms of the Y‐maze are narrow and enclosed by high walls, so this test is unlikely to be confounded by differences in where animals choose to swim/search relative to the side walls of the maze. Data were analysed in three 10‐trial blocks. Genotype had no effect on choice accuracy during the 30‐trial acquisition phase (*F*_1,26_ = 0.721, *P* = 0.404; Fig. 5A). There was a significant main effect of block (*F*_2,52_ = 28.667, *P* < 0.001), but no interaction between genotype and block (*F*_2,52_ = 0.105, *P* = 0.900). Moreover, genotype had no effect on choice accuracy on trial 1 of day 1 (Pearson Chi‐squared test: *χ* ^(1)^ = 0.583, *P* = 0.445). Binomial tests revealed that the proportion of mice that selected the target arm on trial 1 did not differ significantly from chance (i.e. 50%) amongst WT mice (*P* = 1.000) or *Dao* ^−/−^ mice (*P* = 0.424). Although choice accuracy was unaffected, *Dao* ^−/−^ mice took longer than WT mice to reach the escape platform in the Y‐maze (main effect of genotype: *F*_1,26_ = 5.488, *P* = 0.027; Fig. 5B). This difference in escape latency was present from the first trial of the first day (*F*_1,26_ = 5.131, *P* = 0.032).

After 6 days of training (30 trials), a probe test was performed during which the platform was removed and the mice allowed to swim freely for 60 s. The spatial memory performance of the two groups was well matched, with both genotypes showing a clear preference for the target arm (Fig. 5C). Genotype had no effect on time spent in the target arm (*F*_1,25_ = 0.398, *P* = 0.534). Note that one *Dao* ^−/−^ mouse was discounted from these analyses, as it remained motionless for the entirety of the probe trial.

By contrast, mice with hippocampal lesions displayed a profound deficit in the same Y‐maze swim‐escape task, confirming its hippocampal dependency. Data were analysed in five 10‐trial blocks, and there was a main effect of block on choice accuracy (*F*_4,84_ = 11.754, *P* < 0.001; Supporting Information Fig. S4A). More importantly, there was a main effect of lesion (*F*_1,21_ = 104.246, *P* < 0.001), reflecting the inferior performance of the lesioned mice, and a lesion × block interaction (*F*_4,84_ = 6.773, *P* < 0.001), as choice accuracy improved with training in the sham group but not the lesioned group. In addition, the lesioned mice spent less time in the target arm during probe trials performed after 6 days (i.e. 30 trials) of training (*F*_1,21_ = 62.342, *P* < 0.001; Supporting Information Fig. S4B), and after 10 days (i.e. 50 trials) of training (*F*_1,21_ = 62.983, *P* < 0.001; Supporting Information Fig. S4C).

### Experiment 5: normal spatial memory in Dao^−/−^ mice in the appetitively motivated Y‐maze task

**Figure 6:**
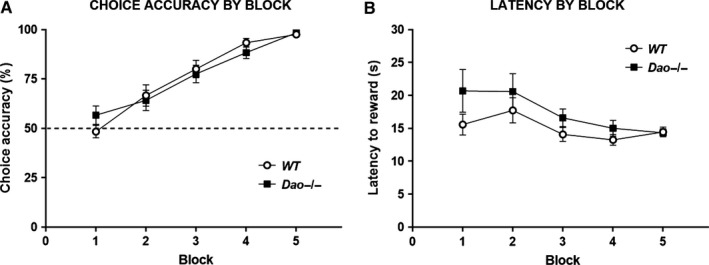
Normal spatial memory in *Dao* ^−/−^ mice in the appetitively motivated Y‐maze task. A total of 24 male mice (*n *= 12 per genotype) participated in Experiment 5. (A) Genotype had no effect on choice accuracy (% correct trials) in this task. (B) Reward latencies were numerically, but not significantly, greater in *Dao* ^−/−^ mice than WT mice. Error bars depict the SEM. Dashed line, chance performance (50%).

Experimentally naive WT and *Dao* ^−/−^ mice were trained on a hippocampus‐dependent, appetitively motivated version of the Y‐maze spatial reference memory task (Deacon *et al*., 2002; Reisel *et al*., 2002). Data were analysed in five 10‐trial blocks. During the 50‐trial acquisition phase, genotype had no effect on choice accuracy (*F*_1,22_ = 0.002, *P* = 0.962; Fig. 6A). There was a significant main effect of block (*F*_4,88_ = 64.164, *P* < 0.001), but no interaction between genotype and block (*F*_4,88_ = 1.262, *P* = 0.291). Furthermore, genotype had no effect on choice accuracy on trial 1 of day 1 (Pearson Chi‐squared test: χ^(1)^ = 1.510, *P* = 0.219). Binomial tests revealed that the proportion of mice that selected the target arm on trial 1 did not differ significantly from chance (i.e. 50%) amongst WT mice (*P* = 0.388) or *Dao* ^−/−^ mice (*P* = 0.774). In general, *Dao* ^−/−^ mice took slightly longer than WT mice to reach the milk reward, although this difference was not statistically significant (*F*_1,22_ = 1.954, *P* = 0.176; Fig. 6B).

To eliminate the possibility that mice were selecting which arm to enter based on odour cues from the milk, a total of 10 post‐choice bait trials were conducted over a further 2 days of testing, in which the milk was only added to the food‐well after the animal had made its choice. During these trials, average choice accuracy remained high in both WT mice (*M* = 98.3%) and *Dao* ^−/−^ mice (*M* = 95.0%), confirming that they were not solving the task by smelling the milk reward.

## Discussion

In the present study, we examined long‐term spatial memory in *Dao* ^−/−^ and WT mice. *Dao* ^−/−^ mice outperformed their WT littermates in the Morris water maze when the hidden platform was located in the periphery of the pool, but were outperformed by WT mice when the platform was positioned in a more central location. These results probably reflect the fact that *Dao* ^−/−^ mice spent more time in the periphery of the pool than WT mice, rendering them more likely to encounter the peripheral platform by chance, but less likely to discover the central platform. The altered swim/search behaviour of *Dao* ^−/−^ mice may be a consequence of heightened anxiety, as they also demonstrated an increased preference for the periphery of a brightly lit, open field arena, an established indicator of anxiety (see also Pritchett *et al*., 2015). Long‐term spatial memory performance was unaffected by *Dao* knockout in both the aversive and appetitive Y‐maze tasks, two alternative assays of hippocampus‐dependent long‐term spatial memory. Hence, in contrast to previous reports of enhanced water maze acquisition in ddY/*Dao* ^−^ mice (Maekawa *et al*., 2005), and contrary to predictions based on the NMDAR‐dependent synaptic plasticity/memory hypothesis, long‐term spatial memory is unaltered in *Dao* ^−/−^ mice.

### Relationship to other rodent models and therapeutic implications

It has been suggested that DAO inhibition could improve cognition by increasing synaptic D‐serine levels and thereby increasing the activation of NMDARs (Smith *et al*., 2010; Ferraris & Tsukamoto, 2011; Sacchi *et al*., 2013). Although the present findings question whether DAO inactivation leads to a genuine enhancement of associative, long‐term spatial memory, there is now a reasonable body of evidence linking DAO inactivation with enhancements in other memory domains. We recently observed improved short‐term memory performance in *Dao* ^−/−^ mice, in both spatial and non‐spatial tasks (Pritchett *et al*., 2015). Likewise, short‐term spatial memory is facilitated in WT mice following the administration of exogenous D‐serine (Bado *et al*., 2011). In addition, exogenous D‐serine and DAO inhibitors both improve object recognition memory performance in WT rodents (Hashimoto *et al*., 2008; Karasawa *et al*., 2008; Smith *et al*., 2009; Bado *et al*., 2011; Hopkins *et al*., 2013), although negative results have also been reported with DAO inhibitors in this task (Smith *et al*., 2009). That specific memory domains are selectively affected by the manipulation of DAO is not altogether surprising, given that short‐term and long‐term memory are dissociable processes with distinct underlying neural substrates (e.g. Sanderson *et al*., 2009), as are spatial and non‐spatial memory (e.g. Brown & Aggleton, 2001).

Encouragingly, improved cognition was recently observed in both patients with schizophrenia and dementia in early clinical trials involving the administration of the DAO inhibitor sodium benzoate (Lane *et al*., 2013; Lin *et al*., 2014). Sodium benzoate significantly improved processing speed in the patients with schizophrenia, in addition to visual learning and memory (Lane *et al*., 2013). The latter was assessed with the visual reproduction subtests of the Wechsler Memory Scale Wechsler (1997), which assay short‐term, non‐spatial memory. In sum, DAO inhibition warrants further investigation as a treatment for impairments in certain aspects of cognition.

### Implications for the Morris water maze task

Attempts to understand the neural mechanisms underlying learning and memory, and, by extension, to identify cognitive‐enhancing drugs, have relied heavily on the Morris water maze task (see D'Hooge & De Deyn, 2001; Vorhees & Williams, 2006). It is difficult to overstate the importance and influence of this task, in both academia and industry. Since its introduction in the early 1980s (Morris, 1984), it has been used to evaluate the impact of countless genetic, pharmacological, environmental and lesion‐based manipulations on rodent spatial memory. However, the procedural simplicity of the task belies the complexity of the underlying behavioural processes. Consequently, there are numerous possible explanations for enhanced or impaired water maze performance, not all of which are directly related to memory. For example, differences in sensorimotor abilities, motivation and emotionality can all affect performance in the water maze (see Keith & Rudy, 1990; Saucier & Cain, 1995; D'Hooge & De Deyn, 2001; Bannerman *et al*., 2006; Vorhees & Williams, 2006), and it is notoriously difficult to control for all of these potential confounds.

Our results have a number of implications for the design and interpretation of Morris water maze experiments, which may counteract some of the aforementioned confounds. Firstly, our data suggest that the systematic variation (and counterbalancing) of radial platform distance might be a useful addition to many studies. Although it is generally accepted as best practice to train different animals to different platform locations as defined by the quadrant of the pool in which they are located (e.g. NW vs. SE, and to counterbalance this across groups), it is normally the case that all animals are trained to locate platforms positioned a set radial distance from the side wall. Our data demonstrate how the use of a single radial platform distance could unintentionally bias the outcome of a study. It remains to be seen whether the water maze performance of other transgenic mouse models is equally sensitive to alterations in radial platform distance. It will also be important to determine whether the effects of putative cognitive‐enhancing drugs on water maze performance are dependent on the distance of the platform from the side wall of the pool.

Secondly, our data emphasize the utility of employing a battery of hippocampal‐dependent tasks (including the Morris water maze) when characterizing the spatial memory performance of a mouse model. As different tasks are subject to different confounds, this approach should lead to more reliable conclusions than the use of a single task. Having said this, different spatial memory tasks are likely to recruit distinct psychological processes, and therefore will be subserved by different neurobiological substrates (e.g. Bannerman *et al*., 2012). To the best of our knowledge, neither ddY/*Dao* ^−^ nor *Dao1* ^*G181R*^ mice have been subjected to any tests of long‐term spatial memory apart from the Morris water maze, with the exception of ddY/*Dao* ^−^ mice in the Barnes maze (Zhang *et al*., 2011). Interestingly, the authors of that study noted that the enhanced Barnes maze performance of the *Dao* mutants may have been ‘because of (their) increased edge activity rather than better spatial learning and memory’ (Zhang *et al*., 2011, p. 86; i.e. the mutant mice spent more time in the periphery of the apparatus where the escape holes were located). This assessment strikes a clear parallel with our own observations in the Morris water maze.

Thirdly, the water maze performance of *Dao* ^−/−^ and WT mice in the present study converged across successive trials. This finding underscores the value of analysing Morris water maze data on a trial‐by‐trial basis as well as on a session‐by‐session basis. Collapsing data across the multiple trials could mask effects that are only evident in the first trial of each session, and this possibility becomes more likely the more trials that are performed in each session. This may have contributed to the inconsistent findings concerning the water maze acquisition of *Dao* mutant mice, as the (Maekawa *et al*., study (2005; which reported a significant main effect of genotype) employed only three trials per session whereas the Labrie *et al*., study (2009; in which genotype had no effect) included four trials per session.

Lastly, our data highlight the potential impact of sex on Morris water maze performance. Male rodents tend to outperform females in the water maze, although this effect can be eradicated with the inclusion of a pre‐training phase (Perrot‐Sinal *et al*., 1996; Beiko *et al*., 2004). Sex differences in the water maze have been attributed to variations in levels of corticosterone and oestradiol (Beiko *et al*., 2004; Snihur *et al*., 2008), the fact that males and females favour different swim/search strategies (e.g. Roof & Stein, 1999), and the differential expression of specific transcription factors and splicing factors (see Antunes‐Martins *et al*., 2007; Mizuno & Giese, 2010). In Experiment 2 of the present study, the interaction between genotype and radial platform distance was present in males but not females, resulting in a significant three‐way interaction between genotype, radial platform distance and sex for path length. Notably, *Dao* ^−/−^ mice of both sexes showed increased peripheral swimming in Experiment 2; there was no genotype × sex interaction for time spent in the peripheral zone of the pool. This might reflect the heightened anxiety phenotype of male and female *Dao* ^−/−^ mice, which we previously reported in multiple paradigms including an open field test (Pritchett *et al*., 2015). What remains unclear, however, is why increased peripheral swimming only impacted on the water maze performance (i.e. path lengths and latencies) of male *Dao* ^−/−^ mice in Experiment 2.

Interestingly, sex differences may also have contributed to the previous contradictory findings regarding the water maze acquisition of *Dao* mutants, as the former study (which revealed a significant main effect of genotype) included only male mice (Maekawa *et al*., 2005), whereas the latter study (in which genotype had no effect) employed males and females (Labrie *et al*., 2009). Other procedural differences between these studies are listed in Table S1.

## Conclusions

We have demonstrated that Morris water maze performance in *Dao* ^−/−^ mice is critically dependent on the radial distance of the hidden platform from the side wall. This may reflect innate differences in the swimming behaviour of *Dao* ^−/−^ mice, rather than altered long‐term spatial memory. Our results have implications for (i) understanding the role of DAO in brain function and dysfunction, (ii) the search for cognitive‐enhancing drugs that indirectly modulate NMDARs, and, more generally, (iii) the design and interpretation of Morris water maze experiments.

## Supporting information

Fig. S1. Morris watermaze performance of Dao^−/−^ mice in Experiment 1.
Fig. S2. Morris watermaze performance of Dao^−/−^ mice in Experiment 2.
Fig. S3. Sex differences in the Morris watermaze performance of Dao^−/−^ mice in Experiment 2.
Fig. S4. Impaired spatial memory in wildtype mice with hippocampal lesions in the aversively‐motivated Y‐maze swim‐escape task.
Table S1. Methodological differences between previous studies of Morris watermaze performance in two related Dao mutants.
