# Supplementary material for: Searching for cognitive enhancement in the Morris water maze: better and worse performance in D‐amino acid oxidase knockout (Dao −/−) mice
Source: Eur J Neurosci. 2016 Mar 23;43(7):979–89. doi: 10.1111/ejn.13192 (PMC4855640; doi:10.1111/ejn.13192)
Supplement: Supplementary file 1 — Fig. S1. Morris watermaze performance of Dao−/− mice in Experiment 1. Fig. S2. Morris watermaze performance of Dao−/− mice in Experiment 2. Fig. S3. Sex differences in the Morris watermaze performance of Dao−/− mice in Experiment 2. Fig. S4. Impaired spatial memory in wildtype mice with hippocampal lesions in the aversively‐motivated Y‐maze swim‐escape task. Table S1. Methodological differences between previous studies of Morris watermaze performance in two related Dao mutants. [file EJN-43-979-s001.pdf]

Supplementary Information

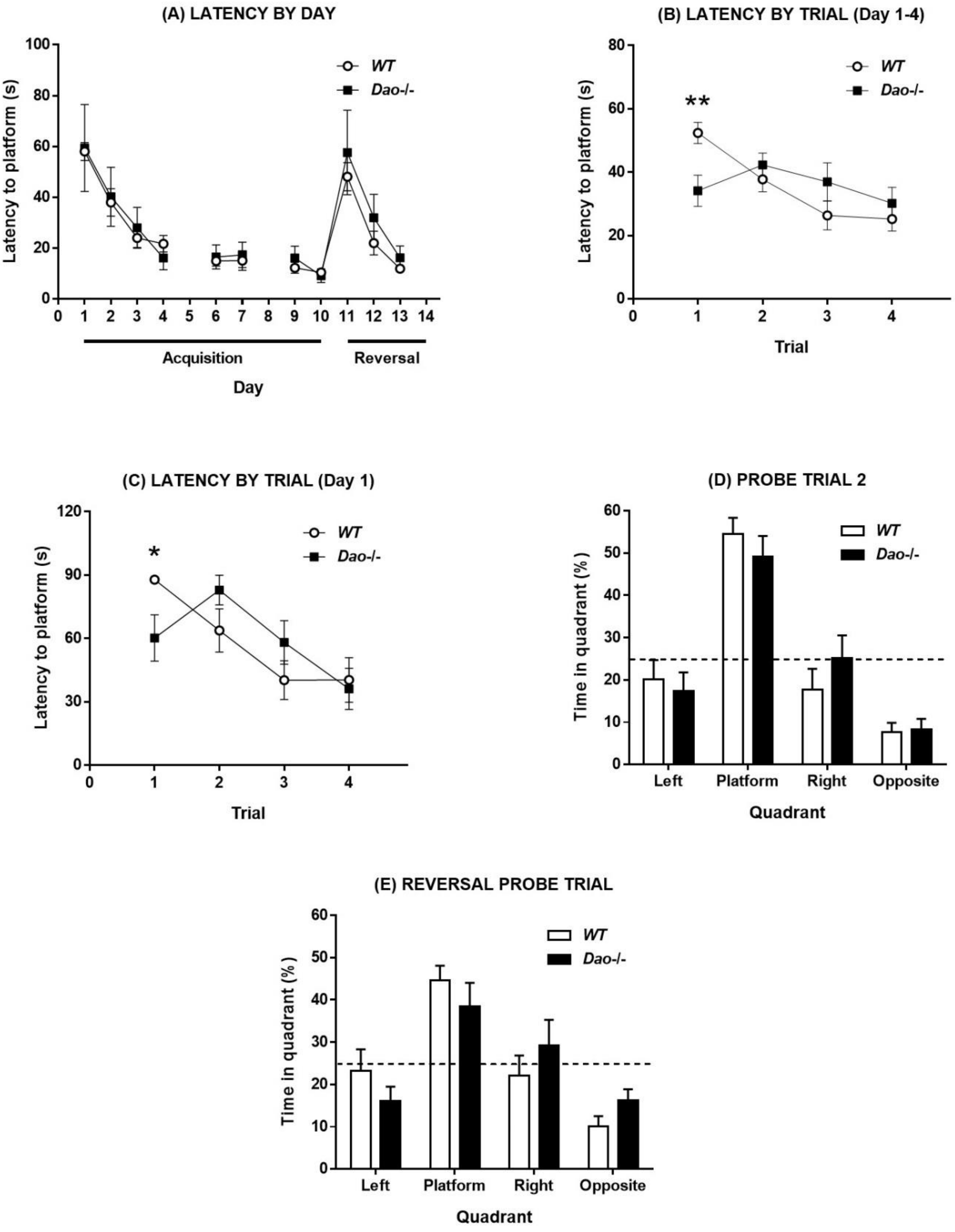

Supplementary Figure 1. Morris watermaze performance of ***Dao***$^{-/-}$ ***mice in Experiment 1.*** **(A) With data collapsed across the four trials of each training session, genotype had no apparent impact on watermaze performance (latency to platform) during the acquisition phase or reversal learning phase. (B) Across the first four days of training, however, *Dao*$^{-}$**

/- mice significantly outperformed WT mice on the first trial of each day (latency to platform), but not on the subsequent three trials, yielding a significant interaction between genotype and trial number (*P* = 0.002). Graph shows latency to the platform in the four trials of a session (Day), collapsed across Days 1-4. (C) The same pattern of performance was evident on the very first day of training, again yielding a significant genotype × trial number interaction (*P* = 0.033). Graph shows pathlength to the platform in the four trials of Day 1. (D) Genotype had no effect on probe trial 2 performance. Graph shows percent time spent in each quadrant of the pool (Platform = quadrant in which the platform was located during training; other quadrants defined relative to this quadrant). (E) Genotype had no effect on reversal probe trial performance (% time in each quadrant). * indicates a p-value < 0.05, ** indicates a p-value < 0.01. Error bars depict the standard error of the mean. Broken line = chance performance (25%).

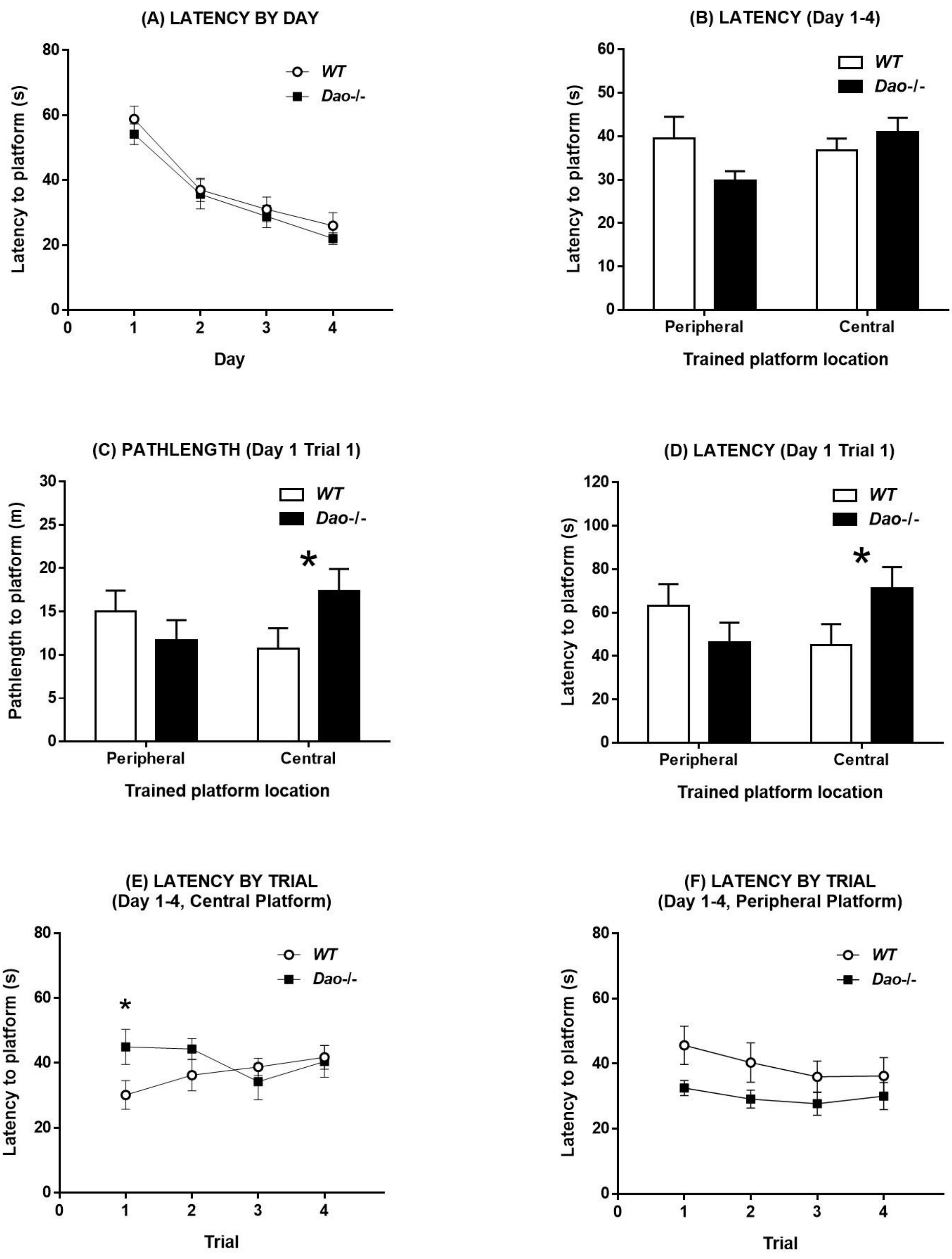

Supplementary Figure 2. Morris watermaze performance of Dao$^{-/-}$ mice in Experiment 2. **(A) With data collapsed across the four trials of each training session, and across the two radial platform distances, genotype had no apparent impact on watermaze performance (latency to platform) during the 4-day acquisition phase. (B) The performance (mean latency to platform across all 16 acquisition trials) of *Dao$^{-/-}$* mice was numerically superior to that of WT mice in the peripheral platform group, but numerically inferior in the**

central platform group, yielding a significant interaction between genotype and radial platform distance (*P* = 0.046). (C-D) A similar pattern of performance was evident on the very first trial of the very first day of training, yielding significant genotype × trial number interactions for both pathlength to platform (*P* = 0.031) and latency to platform (*P* = 0.025). (E) Across the four days of training, WT mice in the central platform condition significantly outperformed *Dao*$^{-/-}$ mice on trial 1 (latency to platform), but not on the subsequent three trials, yielding a significant interaction between genotype and trial number (*P* = 0.035). (F) By contrast, there was no genotype × trial number interaction for latency to platform in the peripheral platform condition. * indicates a p-value < 0.05. Error bars depict the standard error of the mean.

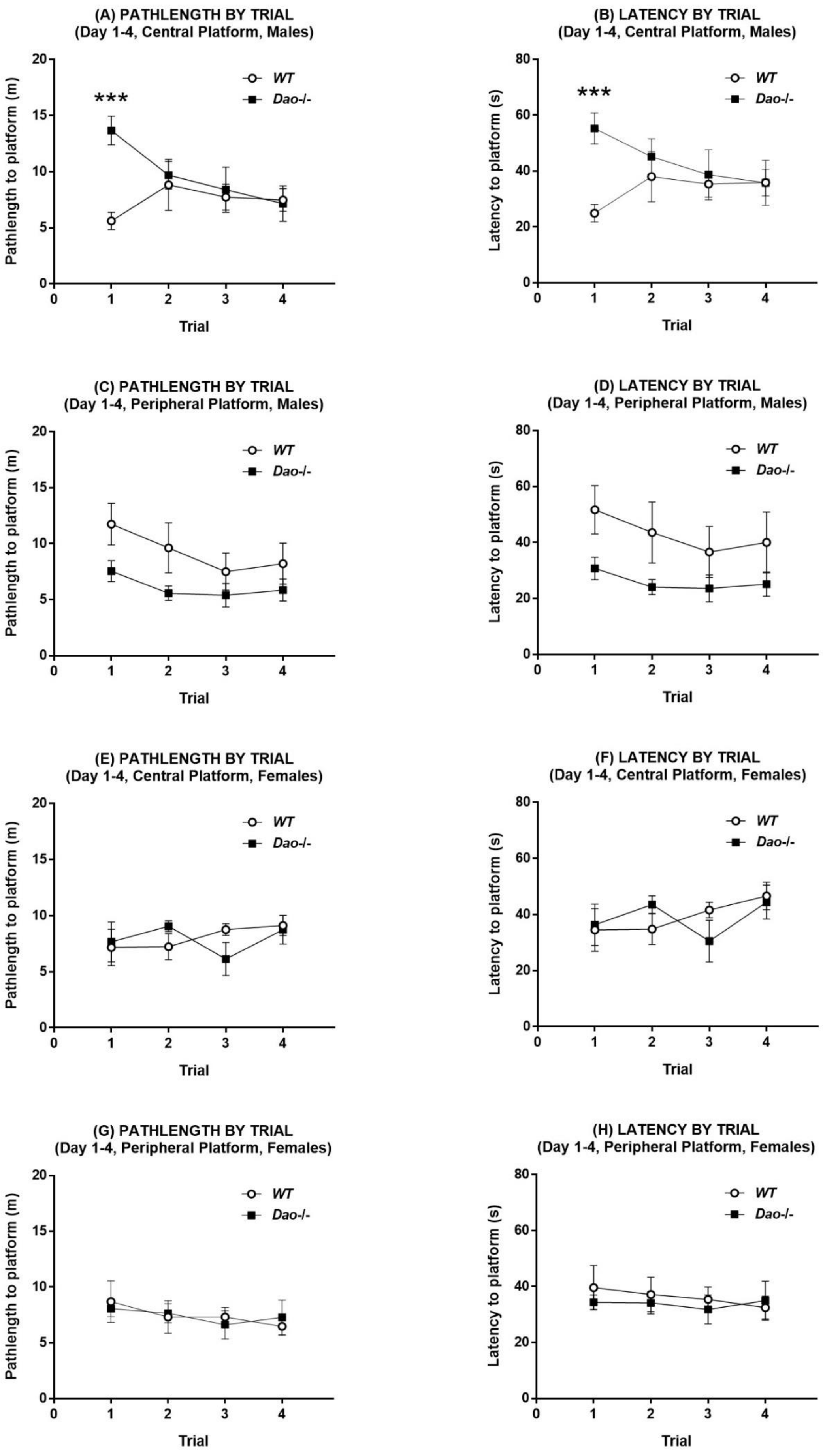
(A) PATHLENGTH BY TRIAL
(Day 1-4, Central Platform, Males)
Pathlength to platform (m)
***
WT
Dao-/-
Trial
(B) LATENCY BY TRIAL
(Day 1-4, Central Platform, Males)
Latency to platform (s)
***
WT
Dao-/-
Trial
(C) PATHLENGTH BY TRIAL
(Day 1-4, Peripheral Platform, Males)
Pathlength to platform (m)
WT
Dao-/-
Trial
(D) LATENCY BY TRIAL
(Day 1-4, Peripheral Platform, Males)
Latency to platform (s)
WT
Dao-/-
Trial
(E) PATHLENGTH BY TRIAL
(Day 1-4, Central Platform, Females)
Pathlength to platform (m)
WT
Dao-/-
Trial
(F) LATENCY BY TRIAL
(Day 1-4, Central Platform, Females)
Latency to platform (s)
WT
Dao-/-
Trial
(G) PATHLENGTH BY TRIAL
(Day 1-4, Peripheral Platform, Females)
Pathlength to platform (m)
WT
Dao-/-
Trial
(H) LATENCY BY TRIAL
(Day 1-4, Peripheral Platform, Females)
Latency to platform (s)
WT
Dao-/-
Trial

Supplementary Figure 3. Sex differences in the Morris watermaze performance of Dao$^{-/-}$ mice in Experiment 2. **(A-B) Across the four days of training, male WT mice in the central platform condition significantly outperformed male *Dao*$^{-/-}$ mice on trial 1, but not on the subsequent three trials, yielding significant interactions between genotype and trial number for both pathlength to platform (*P* = 0.011) and latency to platform (*P* = 0.028). (C-D) By contrast, there were no genotype × trial number interactions for male mice in the peripheral platform condition, for either pathlength or latency to the platform. (E-H) Across the four days of training, genotype had no effect on the performance (pathlength or latency to the platform) of female mice on any trial, in either the central or the peripheral platform condition. *** indicates a p-value < 0.001. Error bars depict the standard error of the mean.**

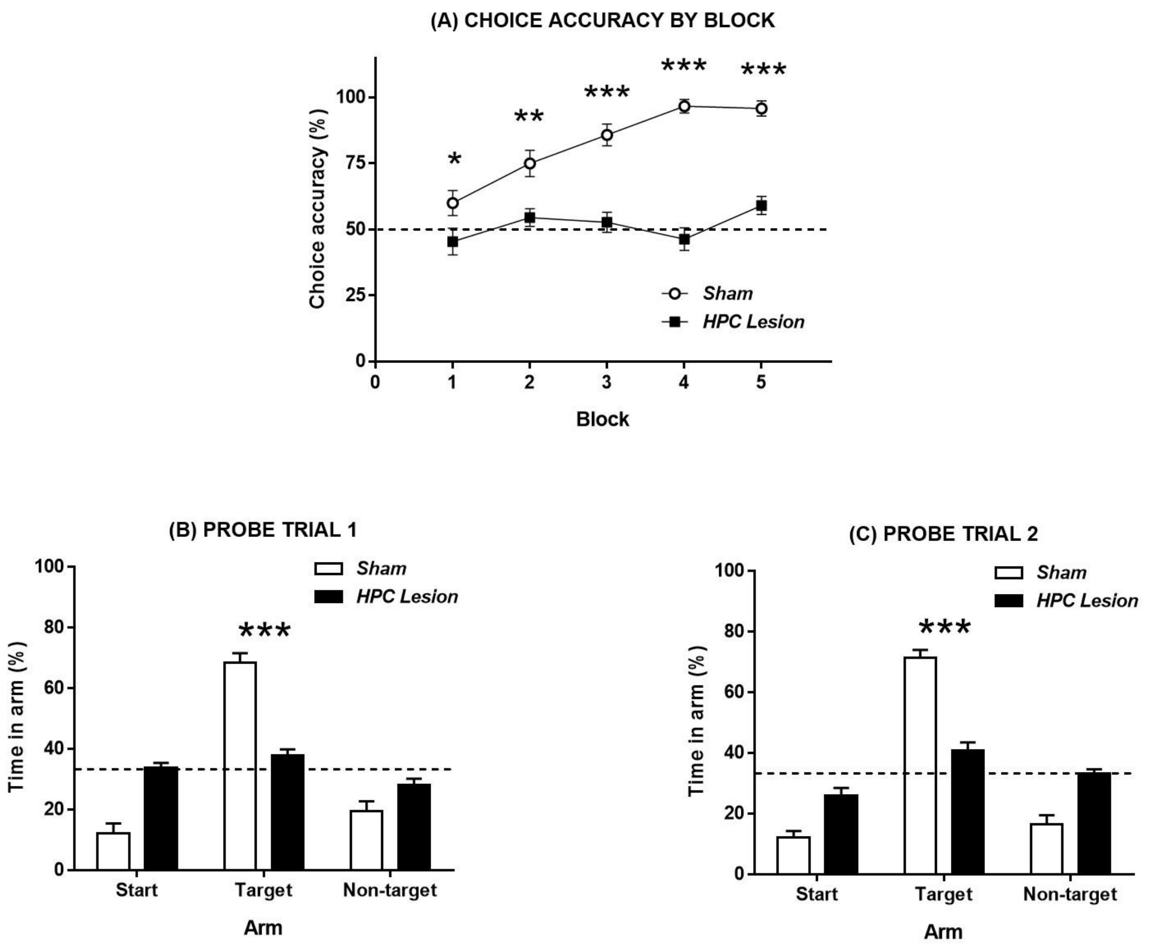

Supplementary Figure 4. Impaired spatial memory in wildtype mice with hippocampal lesions in the aversively-motivated Y-maze swim-escape task. **23 mice (*n*=12 sham females; *n*=11 lesioned females) participated in this experiment. Mice were lesioned at 8 weeks of age, and left to recover for 3 weeks prior to testing. All mice completed 5 training trials per day on days 1-6 and 8-11, and a 30 s probe trial on days 7 and 12. Escape latencies were not recorded. (A) During the acquisition phase, the choice accuracy (% correct trials) of mice with hippocampal lesions was significantly worse than that of sham mice, yielding a significant main effect of lesion (P = < 0.001), and a significant lesion × block interaction (P = < 0.001). (B-C) During both probe trials, mice with hippocampal lesions spent significantly less time in the target arm than sham mice (both *P*s = < 0.001). Graph shows percent time spent in each arm of the maze. (Target = arm in which the platform was located during training). * indicates a p-value < 0.05, ** indicates a p-value < 0.01, and *** indicates a p-value < 0.001. Error bars depict the standard error of the mean. Broken line = chance performance (50% for acquisition phase and 33.3% for probe trials).**

Parameter	Maekawa *et al.*, 2005	Labrie *et al.*, 2009b
Mutant model	ddY/*Dao*$^{-}$	*Dao1*^{G181R}
Background strain	ddY	C57BL/6J
Age at test (weeks)	8-16	11-16
Pool diameter (cm)	70	185
Radial platform distance (cm)	17.5	25
Training trial duration (s)	120	90
Trials per session	3	4
Sessions per day	2	1
Inter-trial interval (h)	Unspecified	1
Gender(s)	Males only	Males and females
Index of probe trial performance	Percent time in the training (platform) quadrant	Percent time within 5 cm of the platform location

***Supplementary Table 1. Methodological differences between previous studies of Morris watermaze performance in two related Dao mutants*. These studies included ddY/*Dao*$^{-}$ mice (Maekawa *et al.*, 2005) and *Dao1*^{G181R} mice (Labrie *et al.*, 2009), respectively.**
